# Neurological manifestations associated with SARS-CoV-2 infection in pediatric patients: a systematic review

**DOI:** 10.1590/1984-0462/2025/43/2024293

**Published:** 2025-11-14

**Authors:** Bruna de Jesus Santos, Emerson André Negrão do Nascimento, Ludmila Oliveira dos Reis, Júlia Belém Lima, Beatriz Belém Lima, Luciana Fernandes Pastana Ramos

**Affiliations:** a Universidade Federal do Pará, Belém, PA, Brazil.

**Keywords:** Nervous system, COVID-19, Child, Infant, Adolescent, Sistema nervoso, COVID-19, Criança, Lactente, Adolescente

## Abstract

**Objective:**

To conduct a systematic review to identify neurological symptoms associated with SARS-CoV-2 in patients aged zero to 19 years, highlighting the main symptoms and addressing the existing gap in age range coverage in current studies.

**Data source:**

This study was registered in the International Prospective Register of Systematic Reviews — PROSPERO (CRD42024520151) and adhered to Preferred Reporting Items for Systematic Reviews and Meta-Analyses — PRISMA (2020) guidelines. Observational and interventional studies, including randomized clinical trials, investigating neurological manifestations in children and adolescents with confirmed SARS-CoV-2 infection were included. Searches were conducted in the United States National Library of Medicine/Medical Literature Analysis and Retrieval System Online (PubMed/MEDLINE), Cochrane Library, and Virtual Health Library (VHL) using Health Science Descriptors/Medical Subject Headings (DeCS/MeSH) terms in English, Spanish, and Portuguese, covering January 2020 to January 2024.

**Data synthesis:**

The search identified 1283 records, of which 302 were excluded (outside of scope), 688 were removed after title/abstract screening, and 95 duplicates were discarded. Fulltext analysis of 198 articles resulted in the selection of 25 eligible studies. The most frequently reported neurological manifestations included seizures, headache, altered levels of consciousness, olfactory and gustatory disturbances, encephalopathy, and acute cerebrovascular diseases.

**Conclusions:**

Neurological manifestations of COVID-19 in children are relatively common, ranging from mild symptoms such as headache and taste/smell disturbances to severe complications like seizures, stroke, altered consciousness, and encephalopathy. Prevalence varies across studies, underscoring the need for further research to clarify underlying mechanisms.

## RESUMO

**Objetivo:**

Analisar os sintomas neurológicos associados ao SARS-CoV-2 em pacientes de zero a 19 anos, identificando os principais sintomas e abordando a lacuna existente na abrangência etária dos estudos atuais.

**Fontes de dados:**

Estudo registrado no International Prospective Register of Systematic Reviews — PROSPERO (CRD42024520151), segundo as diretrizes Preferred Reporting Items for Systematic Reviews and Meta-Analyses — PRISMA (2020). Foram incluídos estudos observacionais e intervencionais, entre os quais ensaios clínicos randomizados, que investigaram manifestações neurológicas em crianças e adolescentes com infecção confirmada por SARS-CoV-2. Descritores em Ciências da Saúde (DeCS) e Medical Subject Headings (MeSH) foram utilizados nas buscas realizadas nas bases de dados Biblioteca Nacional de Medicina dos Estados Unidos/ Sistema Online de Busca e Análise de Literatura Médica (PubMed/MEDLINE), Cochrane Library e Biblioteca Virtual em Saúde (BVS), nos idiomas inglês, espanhol e português, entre janeiro/2020 e janeiro/2024.

**Síntese dos dados:**

A busca resultou em 1.283 registros, sendo 302 excluídos (fora do escopo), 688 desconsiderados após a triagem de título e resumo e 95 duplicatas removidas. A análise de texto completo de 198 artigos levou à seleção de 25 estudos. As manifestações neurológicas mais frequentemente relatadas nos estudos incluídos foram convulsões, dor de cabeça, alterações no nível de consciência, distúrbios olfatórios e gustativos, encefalopatia e doenças cerebrovasculares agudas.

**Conclusões:**

As manifestações neurológicas da COVID-19 em crianças são relativamente comuns, variando de sintomas leves, como cefaleia e distúrbios no paladar e olfato, até complicações graves, incluindo convulsões, acidente vascular cerebral, alteração da consciência e encefalopatia. A prevalência dessas manifestações varia entre os estudos, destacando a necessidade de mais pesquisas para entender melhor os mecanismos subjacentes.

## INTRODUCTION

 The coronavirus disease 2019 (COVID-19) pandemic imposed a significant global burden, necessitating urgent interventions to control the spread of the virus.^1-4^ Severe acute respiratory syndrome coronavirus 2 (SARS-CoV-2) affects all age groups and exhibits a high rate of human-to-human transmission, with each infected individual potentially spreading the virus to an average of 2.2 others.^2^

 Initial cases were identified in December 2019, with the first polymerase chain reaction (PCR) tests for COVID-19 diagnosis becoming available in Wuhan on January 11, 2020.^2^ Pediatric COVID-19 cases show a distinct demographic pattern, with 49% of cases occurring in children aged between one and ten years, and 55% being male.^1^

 Most pediatric cases are asymptomatic or present with mild symptoms such as fever, cough, fatigue, mild respiratory symptoms, and gastrointestinal symptoms.^1,3^ The incidence of severe cases requiring hospitalization, and the mortality rate are significantly lower in children than in adults. However, severe cases, although rare, can lead to significant complications, including neurological and other systemic manifestations.^1,3,4^

 Coronaviruses are known to cause a broad range of neurological symptoms, both acute and chronic. COVID-19 has demonstrated a wider spectrum and higher frequency of neurological symptoms, possibly due to its high transmissibility and global impact.^5,6^ These symptoms range from mild manifestations, such as headaches and myalgia, to more severe complications, including seizures, encephalopathy, strokes, and Guillain-Barré syndrome.^5-12^ Mild symptoms are often among the first signs of infection, while severe neurological manifestation, although less common, represent serious complications and may result in chronic symptoms.^7^ These findings underscore the need for a comprehensive clinical approach to managing post-infection neurological and psychological effects.^5,7,10^

 Despite the relatively low mortality rate, COVID-19 should be considered a significant threat to child and adolescent health.^4^ The neurological symptoms associated with COVID-19 impact quality of life and require continuous multidisciplinary care (neurologists, psychologists, physical therapists, and nutritionists), resulting in significantly higher costs for both patients and healthcare systems.^5-9,11^

 This manuscript aims to conduct a systematic review to identify the neurological symptoms associated with SARS-CoV-2 infection in pediatric patients, addressing an existing gap in the literature. Previous studies often focus on specific age groups — such as the investigation by Aldè et al.^13^ involving children aged five to 11 years — or on isolated manifestations, such as olfactory dysfunctions,^14^ or on severe neurological conditions, including encephalopathy and seizures,^15^ without exploring the full spectrum of presentations. In this context, the present review seeks to provide a comprehensive analysis encompassing all pediatric age groups and the diversity of neurological manifestations reported in the literature. This approach contributes to a broader understanding of the neurological impact of COVID-19 in children and adolescents, offering valuable insights for future research. 

## METHOD

 The protocol for this systematic review was registered and published on the International Prospective Register of Systematic Reviews (PROSPERO) under the identifier CRD42024520151. In accordance with the 2020 Preferred Reporting Items for Systematic Reviews and Meta-analyses guidelines (PRISMA),^16^ a systematic search was conducted on the United States National Library of Medicine/Medical Literature Analysis and Retrieval System Online (PubMed/MEDLINE), Cochrane Library, and Biblioteca Virtual em Saúde (BVS), from database inception to January 2024. 

 The review questions guiding this proposal were formulated using the Population, Exposure, Controls, and Outcomes (P.E.C.O.) strategy, as shown in Table 1. 

**Table 1:** Population, Exposure, Control, Outome strategy for formulating the guiding question.

P	Pediatric patients.
E	Sars-Cov-2 virus infection.
C	The studies can or cannot be compared.
O	Identify the main neurological manifestations.

P: Population; E: Exposure; C: Control; O: Outome.

 The search strategy was developed and iteratively refined to ensure both comprehensive coverage and accuracy, utilizing the following terms: (Neurologic Manifestation OR Neurologic Manifestations OR Neurological Manifestation OR Neurological Manifestations OR Neurologic Signs and Symptoms OR Neurologic Deficits OR Neurologic Deficit OR Neurologic Symptoms OR Neurologic Symptom OR Neurologic Findings OR Neurologic Finding OR Neurologic Signs OR Neurologic Sign OR Focal Neurologic Deficits OR Focal Neurologic Deficit OR Neurologic Dysfunction OR Neurologic Dysfunctions) AND (2019-nCoV Infection OR 2019-nCoV Infections OR SARSCoV-2 Infection OR SARS-CoV-2 Infections OR 2019 Novel Coronavirus Disease OR 2019 Novel Coronavirus Infection OR COVID-19 Virus Infection OR COVID-19 Virus Infections OR COVID-19 OR Coronavirus Disease-19 OR Severe Acute Respiratory Syndrome Coronavirus 2 Infection OR COVID19 Virus Disease OR COVID-19 Virus Diseases OR SARS Coronavirus 2 Infection OR 2019-nCoV Disease OR 2019nCoV Diseases OR COVID-19 Pandemic OR COVID-19 Pandemics) AND (Child OR Children). No restrictions on language or publication date were applied. 

 Titles and abstracts were independently screened by five review authors (B.S., E.N. L.R., J.B., B.B.) using the software Mendeley Reference Manager (version 2.110.2). Articles identified as potentially relevant by at least one reviewer were retrieved, while duplicates or unrelated to humans were excluded. Full-text articles of potentially eligible studies were reviewed to determine final inclusion or exclusion, with any discrepancies resolved through discussion. 

**Figure 1:**
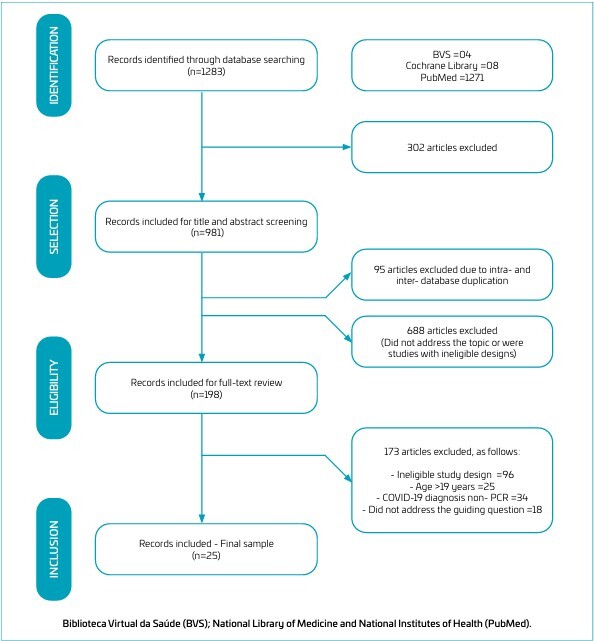
Flow diagram of the study selection process.

 Only studies whose population is children and adolescents will be included. For this investigation, the definition of "children and adolescents" by the World Health Organization (WHO) will be considered, which refers to a person aged between zero and 19 years old.^17^ Eligible studies were those that reported neurological symptoms in patients with a confirmed history of SARS-CoV-2 infection based on polymerase chain reaction (PCR) testing. Both observational and intervention study design were considered, including randomized controlled trials (RCTs) and before-and-after studies without a control group. Only original studies published in peer-reviewed journals were included. The study selection flowchart is presented in Figure 1. 

**Exclusion criteria were:**
1. Non-original studies, such as systematic reviews, narrative reviews, editorials, correspondence, abstracts, and methodological studies;
2. Incomplete or imprecise quantitative data;
3. No report on the measures of interest;
4. Outcomes reported solely in the general population or in individuals without a confirmed prior COVID-19 diagnosis based on PCR testing;
5. Studies without pediatric patients; and
6. Case report.

The data from the included studies were extracted using a pre-designed form in Microsoft Office Excel software (version 2021).

**The form included the following items:**
1. Identification with the first author’s name and year of publication;
2. Study design;
3. Sample size;
4. Location;
5. Age range;
6. Clinical manifestations.

Clinical manifestations were listed in the spreadsheet and correlated based on the identifiers. 

 Methodological quality and risk of bias was assessed using the National Institutes of Health quality assessment tool for the before-and-after study without a control group and corresponding to the Joanna Briggs Institute (JBI) critical appraisal checklists for cross-sectional and RCT study designs (Joanna Briggs Institute).^18^ Three reviewers (B.S., L.R., and E.N.) appraised the studies independently and results were corroborated, with discrepancies resolved through discussion. 

 Following the critical appraisal of the methodological quality of each study, a grading system was applied to determine the final inclusion or exclusion of individual studies. The three study quality levels were: low quality (0 to 33% of criteria met), moderate quality (34 to 66% of criteria met), or high quality (67% or more of criteria met). 

## RESULTS

 A total of 1283 records were initially identified through database searches. Of these, 302 were excluded for not fitting the scope of the review. The remaining 981 articles had their titles and abstracts evaluated, resulting in the exclusion of 688 studies that did not meet the inclusion criteria. Additionally, 95 duplicate records were removed. During the full-text review phase, 198 articles were analyzed in detail, of which 25 studies met all eligibility^ 13,19- 42^ criteria and were therefore included in this review. The entire screening and selection process is detailed in the PRISMA flow diagram (Figure 1). 

 All studies included in this systematic review were observational in nature and involved populations of children aged zero to 19 years. The 25 selected studies were conducted in various countries, including Turkey, Italy, the United States of America, Saudi Arabia, India, Mexico, the United Kingdom, Russia, Denmark, China, and Iran, with publications starting in 2020. The detailed characteristics of the studies, as well as the specificities of the populations evaluated, are presented in Table 2 and 3 .^13,19 -42^

**Table 2:** Characteristics of studies included in our systematic review.

Reference	Locality	Type of study	n	Age group
Abbati et al.^19^	Italy	Retrospective cohort	122	0–18
Aldè et al.^13^	Italy	Retrospective cohort	272	5–11
Alzahrani et al.^20^	Saudi Arabia	Case-control	468	0–14
Borch et al.^21^	Denmark	Prospective cohort	30,121	0–17
Dilber et al.^22^	Turkey	Retrospective descriptive	2530	0–17
Elvan-Tuz et al.^23^	Turkey	Prospective cohort	10,157	10–18
Fang et al.^24^	China	Retrospective descriptive	103	0–5
Flores-Alanis et al.^25^	México	Retrospective descriptive	1349	0–19
Gürkas et al.^26^	Turkey	Prospective descriptive	312	0–18
Hegazi et al.^27^	Saudi Arabia	Retrospective cross-sectional	94	0–19
Kumar et al.^28^	India	Prospective cross-sectional	141	10–19
LaRovere et al.^30^	United States	Prospective cross-sectional	1695	0–20
LaRovere et al.^29^	United States	Retrospective cross-sectional	2168	0–20
López-Pérez et al.^31^	Mexico	Retrospective cohort	46	1–17
Memar et al.^32^	Iran	Retrospective cohort	54	0–18
Okur^33^	Turkey	Retrospective cohort	243	0–18
Pascarella et al.^34^	Italy	Retrospective cohort	504	0–14
Rastogi et al.^35^	India	Retrospective cross-sectional	19	0–12
Ray et al.^36^	United Kingdom	Prospective cohort	52	0–18
Riva et al.^37^	Italy	Prospective cohort	237	0–18
Rusetsky et al.^38^	Russia	Prospective cross-sectional	79	6–17
Salleh et al.^39^	Brunei	Prospective cross-sectional	649	0–12
Stephenson et al.^40^	United Kingdom	Prospective cohort	3395	11–17
Westbrook et al.^41^	United States	Prospective cohort	602	0–18
Yilmaz et al.^42^	Turkey	Retrospective descriptive study	706	0–18

**Table 3:** Characteristics of studies included in our systematic review.

Reference	Neurological manifestations
Abbati et al.^19^	Consciousness impairment; Irritability/agitation; Drowsiness/hyporeactivity; Confusion; Temporary loss of consciousness; Stupor/coma; Headaches; Seizures; Behavioral changes; Mood disorders; Anxiety disorders; Photophobia; Meningeal signs; Protruding fontanelle; Dizziness; Dysgeusia/ageusia; Hyper/hypotonia; Balance issues; Gait disturbances; Motor deficits; Retrograde amnesia; Speech disorders; Visual hallucinations; Visual impairment.
Aldè et al.^13^	Vertigo; Dizziness.
Alzahrani et al.^20^	Headache; Dysgeusia; Parosmia.
Borch et al.^21^	Myasthenia; Headache; Ageusia; Anosmia; Dizziness; Difficulty concentrating.
Dilber et al.^22^	Headache; Anosmia/ageusia; Vertigo/nausea; Dizziness; Seizures (febrile, acute symptomatic, and afebrile); Meningoencephalitis.
Elvan-Tuz et al.^23^	Anosmia; Ageusia; Myasthenia; Headache.
Fang et al.^24^	Complex febrile seizure; Recurrent seizure; Focal seizure; Seizures lasting ≥15 minutes; Status epilepticus.
Flores-Alanis et al.^25^	Anosmia; Dysgeusia.
Gürkas et al.^26^	Headache; Anosmia/hyposmia; Ageusia; Vertigo.
Hegazi et al.^27^	Headache; Hallucination; Anosmia/hyposmia; Seizure; Impaired consciousness; Asthenia; Dysarthria; Irritability.
Kumar et al.^28^	Hyposmia; Anosmia; Headache; Dysgeusia.
LaRovere et al.^30^	Severe encephalopathy; Difficulty walking/crawling; Dysgeusia; Seizures; Headache; Hallucinations; Impaired consciousness; Stroke; Central nervous system demyelinating infection; Guillain-Barré syndrome/variants; Fulminant acute cerebral edema.
LaRovere et al.^29^	Confusion; Headache; Dysgeusia/anosmia; Acute central nervous system infection/acute disseminated encephalomyelitis; Guillain-Barré syndrome; Ischemic or hemorrhagic stroke; Severe encephalopathy; Fulminant acute cerebral edema; Guillain-Barré syndrome; Focal seizure or epileptic discharge.
López-Pérez et al.^31^	Headache; Encephalopathy; Seizures; Neuropathy; Cerebral Hemorrhage; Subarachnoid Hemorrhage.
Memar et al.^32^	Anosmia; Ageusia; Strabismus; Headache; Vision disorders; Vertigo; Temporary loss of consciousness; Seizure; Behavioral/personality changes; Myasthenia; Ataxia; Stroke; Focal neurological disorders; Movement disorders.
Okur^33^	Anosmia/hyposmia; headache.
Pascarella et al.^34^	Seizure.
Rastogi et al.^35^	Movement disorders; Behavioral changes; Sensory changes; Guillain-Barré syndrome; Encephalopathy; Acute flaccid paralysis; Dysphagia; Hemiparesis; Encephalitis; Cerebrovascular event/stroke; Seizures.
Ray et al.^36^	Encephalopathy; Seizures; Headache/meningism; Behavioral changes; Hallucinations; Encephalitis; Epilepsy; Meningitis; Acute Demyelinating Syndrome; Acute psychosis; Chorea; Stroke; Diffuse myopathic or neuropathic changes; Primary demyelinating polyneuropathy; Right fibular and tibial neuropathy; Proximal myopathy and bilateral tibial neuropathies; Unilateral facial nerve injury; Guillain-Barré syndrome.
Riva et al.^37^	Headache; Altered consciousness; Ageusia/anosmia; Seizure; Vertigo; Intracranial hypertension; Facial paralysis; Nuchal rigidity.
Rusetsky et al.^38^	Hyposmia.
Salleh et al.^39^	Febrile seizure; Dizziness; Lethargy.
Stephenson et al.^40^	Headache; anosmia; ageusia; dizziness/fainting; confusion/disorientation/drowsiness.
Westbrook et al.^41^	Headache; Anosmia; Dysgeusia.
Yilmaz et al.^42^	Headache; Dysgeusia; Anosmia; Dementia.

### Headache

 Among the 25 studies included in this review, 18 identified headache as a neurological manifestation, with ten cohort studies, three descriptive studies, four cross-sectional studies, and one case-control study. Hegazi et al. reported neurological manifestations associated with COVID-19 in 29 patients (30.9%), with headache being the most common symptom (27.6%).27 Similarly, in a cohort study by López-Pérez et al., of 46 children hospitalized with confirmed SARS-CoV-2 infection, 23 exhibited neurological manifestations, headache being the most common (56%).^31^

### Taste disorders

 A total of 16 studies reported taste disorders as a neurological manifestation in pediatric patients. Of these, nine were cohort studies, three were descriptive studies, three were cross-sectional studies, and one was a case-control study. Alzahrani et al., in their case-control study, demonstrated that, among 234 children with COVID-19, 24% exhibited taste disturbances.^20^

### Olfactory disorders

 Olfactory disorders were identified as a neurological manifestation in 16 studies, including nine cohort studies, four cross-sectional studies, and three descriptive studies. In the study by Elvan-Tuz et al., among 10,157 pediatric patients aged ten to 18 years who tested positive for COVID-19, 12.5% presented with anosmia.^23^

### Seizure

 Among the studies included in this review, 12 reported febrile or afebrile seizures as a significant manifestation in children with COVID-19. Of these, six were retrospective cohort studies, two prospective cross-sectional studies, three were retrospective cross-sectional studies, and one was a retrospective descriptive study. Dilber et al. highlighted that seizures were the leading cause of hospitalization among neurological complications.^22^ This underscores the clinical significance of seizures (with or without fever), as demonstrated by Pascarella et al. cohort, where approximately 18% of the 504 participating children experienced seizures, with 82.8% of these cases presenting seizures as the sole manifestation of SARS-CoV-2 infection.^34^

### Encephalopathies

 Encephalopathies, defined as any diffuse brain function disorder with acute onset, were reported in five studies: two retrospective cross-sectional studies, one prospective cross-sectional study, one retrospective cohort study, and one prospective cohort study. In the retrospective study by Rastogi et al.,^35^ which analyzed children with neurological manifestations associated with COVID-19, encephalopathy was identified as the second most common neurological condition in this population, corroborating the findings of López-Pérez et al.^31^

### Impaired consciousness

 Impaired consciousness was described in five studies: two retrospective cohort studies, one1 retrospective cross-sectional study, one prospective cross-sectional study, and one prospective cohort study. According to Hegazi et al., in their cross-sectional study, neurological manifestations associated with COVID-19 were found in 29 patients (30.9%), headache being the most common symptom (27.6%), followed by impaired consciousness (20.7%).^27^

### Acute cerebrovascular disease

 Acute cerebrovascular diseases, such as ischemic stroke, cerebral hemorrhage, and subarachnoid hemorrhage, were observed in six studies included in this review, consisting of three cohort studies and three cross-sectional studies. LaRovere et al., in their retrospective cross-sectional study, demonstrated that 476 children exhibited neurological involvement, with 42 in critical condition, and, of these, 11 presented with stroke.^29^

### Quality assessment

 Assessment of the methodological quality and risk of bias of the included studies was conducted using the JBI instrument, following the 2017 guidelines.^18^ The results of this assessment, which address the robustness of the methods and the presence of potential biases, are detailed and presented in Table 4. 

**Table 4:** Summary of risk of bias.

Cohort studies														
References	D1	D2	D3	D4	D5	D6	D7	D8	D9	D10	D11	n (%)	Quality	Risk of bias
Abbati et al.^19^												6 (54.5)	Medium	Moderate
Aldè et al.^13^												9 (81.8)	High	Low
Borch et al.^21^												4 (36.3)	Low	High
Dilber et al.^22^												6 (54.5)	Medium	Moderate
Elvan-Tuz et al.^23^												7 (63.6)	Medium	Moderate
López-Pérez et al.^31^												11 (100)	High	Low
Memar et al.^32^												5 (45.4)	Medium	Moderate
Pascarella et al.^34^												8 (72.7)	High	Low
Ray et al.^36^												6 (54.5)	Medium	Moderate
Riva et al.^37^												6 (54.5)	Medium	Moderate
Okur^33^												10 (90.9)	High	Low
Stephenson et al.^40^												8 (72.7)	High	Low
Westbrook et al.^41^												8 (72.7)	High	Low
Cross-sectional studies														
References	D1	D2		D3	D4	D5		D6	D7		D8	n (%)	Quality	Risk of bias
Hegazi et al.^27^												7 (87.5)	High	Low
Kumar et al.^28^												4 (50)	Medium	Moderate
LaRovere et al.^30^												7 (87.5)	High	Low
LaRovere et al.^29^												6 (75)	High	Low
Rastogi et al.^35^												4 (50)	Medium	Moderate
Rusetsky et al.^38^												7 (87.5)	High	Low
Rusetsky et al.^39^												8 (100)	High	Low
Case-control studies														
References	D1	D2		D3	D4	D5	D6	D7	D8	D9	D10	n (%)	Quality	Risk of bias
Alzahrani et al.^20^												8 (80)	High	Low
Descriptive studies														
References	D1	D2		D3	D4	D5	D6	D7	D8		D9	n (%)	Quality	Risk of bias
Fang et al.^24^												6 (66.6)	Medium	Moderate
Flores-Alanis et al.^25^												7 (77.7)	High	Low
Gürkas et al.^26^												4 (44.4)	Medium	Moderate
Yilmaz et al.^42^												6 (66.6)	Medium	Moderate

Cohort studies are assessed based on 11 criteria, including recruitment, exposure measurement, confounding control, and follow-up. Cross-sectional studies have 8 criteria, such as sample definition, exposure measurement, and statistical analysis. Case-control studies are evaluated using 10 criteria, including group comparability, exposure measurement, and sufficient study duration. Descriptive studies follow 9 criteria, considering sampling, confounding, and measurement reliability. Study quality is classified as low (0–37%), medium (38–66.9%), or high (≥67%).

## DISCUSSION

 In this systematic review, we identified and described the neurological manifestations associated with SARS-CoV-2 infection in children based on current evidence. The analysis of existing studies allowed for the identification of neurological symptoms linked to COVID-19 in pediatric patients, highlighting the complexity and diversity of these symptoms. 

 The prevalence of neurological manifestations varied significantly across the analyzed studies, ranging from 21.15^29^ to 61.1%.^19^ This phenomenon aligns with findings from previous studies in adults,^43,44^ which also reported a wide range of prevalence rates (36.4 to 57.4%). In children, our review found a higher overall prevalence compared to Panda et al.,^15^ who identified a rate of approximately 16% in their meta-analysis. This discrepancy may be attributed to the inclusion of more recent studies in our review, potentially capturing a broader spectrum of neurological manifestations or reflecting differences in circulating viral variants. 

 Among the identified neurological manifestations, seizures and headaches were the most frequently reported symptoms, although their specific prevalence varied between studies. Seizures were highlighted as a common manifestation in some studies,^19,32^ corroborating observations that seizures appear more frequent in children compared to adults.^34,45^ Headaches were also frequently reported^19,22 ,26,27^ and are considered a symptom with high positive predictive value for COVID-19 diagnosis in children.^41^ Other recurrent neurological symptoms included dizziness, anosmia/hyposmia, ageusia,^23,26 ,28,38^ meningoencephalitis,^22^ and vertigo.^13,26^

 Our findings corroborate those of Jin et al.,^46^ which indicate the potential for neurological involvement even in mild clinical presentations of COVID-19. Additionally, our study revealed a significant subgroup of children with severe neurological involvement characterized by life-threatening complications such as severe encephalopathy, acute ischemic or hemorrhagic stroke, acute disseminated encephalomyelitis, fulminant cerebral edema, and Guillain-Barré syndrome.^29,30 ,35,36^ While 8.9% of patients with neurological complications required hospitalization,^22^ the mortality rate was significantly higher (p<0.025) among children with neurological symptoms compared to those without, underscoring the importance of early recognition and treatment.^31^

 Olfactory and gustatory disorders were more prevalent in COVID-19 patients compared to other upper respiratory tract infections.^23^ Anosmia was particularly associated with milder disease courses; however, recovery rates for this symptom tended to decrease with increasing disease severity due to unclear mechanisms.^23^ This suggests that factors such as host immune response and viral load may play critical roles in the manifestation and recovery of these disorders. 

 Consistent with a systematic review involving 19,424 COVID-19 patients, where anosmia prevalence ranged from 4.23 to 98.33%,^47^ our study also revealed wide variability (1.9 to 86.1%).^26 ,38^ Similarly, taste disorders showed significant variation (0.3 to 84%).^23,26^ These findings highlight substantial heterogeneity in anosmia and taste disorder rates among pediatric populations, potentially reflecting differences in study sample characteristics, infection severity, and symptom evaluation methods. Sensory assessment challenges in young children may also contribute to this variability.^14^

 Altered consciousness emerged as a relevant neurological manifestation in children with COVID-19.^19,27,29,32,37^ Although its exact prevalence varied, studies consistently documented its occurrence among pediatric patients. For example, an American study involving 1,695 children found that approximately 25% of cases with neurological involvement exhibited altered consciousness or confusion regardless of age group.^29^ Hospitalized children showed even higher prevalence rates; one study indicated that about 67.4% demonstrated some level of consciousness impairment.^19^ These differences suggest that impaired consciousness may be an important marker for the severity of COVID-19 in children. 

 Seizures were identified as the leading cause of hospitalization among children with neurological complications.^22^ The presence of comorbidities such as epilepsy and a history of febrile seizures was significantly associated with an increased incidence of this manifestation.^24,34^ Additionally, our review noted altered patterns of febrile seizures typically observed during seasonal viral infections; in the context of COVID-19, febrile seizures appeared to affect older children more frequently than other respiratory viruses.^24^

 This review provides a significant contribution by offering a comprehensive analysis of COVID-19’s neurological manifestations in children aged 0–19 years. Unlike previous reviews focusing on specific age groups or isolated manifestations, our analysis integrates diverse neurological symptoms for a more holistic perspective. The findings have important clinical implications, as early identification of common and severe neurological manifestations can facilitate more effective diagnosis and treatment, improving patient outcomes. 

 Study limitations include high heterogeneity in prevalence estimates for some neurological manifestations, the inclusion of studies with small sample sizes, and the absence of a meta-regression analysis that could elucidate sources of variation. Differences between SARS-CoV-2 variants and vaccination impacts on manifestation occurrence were not explored either. 

 Although there is extensive literature on pediatric COVID-19 manifestations, some relevant studies may have been excluded due to strict eligibility criteria. Most included studies were classified as moderate quality, potentially affecting result robustness. Therefore, there is an urgent need for high-quality prospective cohort studies to confirm that the reported neurological manifestations are not coincidental findings from analyzed studies. 

 As a conclusion, neurological manifestations associated with COVID-19 in pediatric patients are diverse, ranging from mild or nonspecific symptoms, such as headache, anosmia/hyposmia, ageusia, and dizziness, to severe complications, including seizures, altered levels of consciousness, encephalopathy, and cerebrovascular accidents. The prevalence of these manifestations showed significant variability across the analyzed studies, reflecting the heterogeneity of the studied populations and the severity of the infection. Therefore, the conduction of robust, high-quality prospective studies is crucial to confirm these findings and to further elucidate the mechanisms underlying the neurological manifestations of COVID-19 in children. 

## Data Availability

The database that originated the article is available with the corresponding author.

## References

[1] Cui X, Zhao Z, Zhang T, Guo W, Guo W, Zheng J (2021). A systematic review and meta-analysis of children with coronavirus disease 2019 (COVID-19). J Med Virol.

[2] Li Q, Guan X, Wu P, Wang X, Zhou L, Tong Y (2020). Early transmission dynamics in wuhan, china, of novel coronavirus-infected pneumonia. N Engl J Med.

[3] Ludvigsson JF (2020). Systematic review of COVID-19 in children show milder cases and a better prognosis than adults. Acta Paediatr.

[4] Flaxman S, Whittaker C, Semenova E, Rashid T, Parks RM, Blenkinsop A (2023). Assessment of COVID-19 as the underlying cause of death among children and young people aged 0 to 19 years in the US. JAMA Netw Open.

[5] Wu Y, Xu X, Chen Z, Duan J, Hashimoto K, Yang L (2020). Nervous system involvement after infection with COVID-19 and other coronaviruses. Brain Behav Immun.

[6] Picone P, Sanfilippo T, Guggino R, Scalisi L, Monastero R, Baschi R (2023). Neurological consequences, mental health, physical care, and appropriate nutrition in long-COVID-19. Cell Mol Neurobiol.

[7] Lai CC, Ko WC, Lee PI, Jean SS, Hsueh PR (2020). Extra-respiratory manifestations of COVID-19. Int J Antimicrob Agents.

[8] Misra S, Kolappa K, Prasad M, Radhakrishnan D, Thakur KT, Solomon T (2021). Frequency of neurologic manifestations in COVID-19: a systematic review and meta-analysis. Neurology.

[9] Qi K, Zeng W, Ye M, Zheng L, Song C, Hu S (2021). Clinical, laboratory, and imaging features of pediatric COVID-19: a systematic review and meta-analysis. Medicine (Baltimore).

[10] Lopez-Leon S, Wegman-Ostrosky T, Del Valle NC, Perelman C, Sepulveda R, Rebolledo PA (2022). Long-COVID in children and adolescents: a systematic review and meta-analyses. Sci Rep.

[11] Munblit D, Nicholson TR, Needham DM, Seylanova N, Parr C, Chen J (2022). Studying the post-COVID-19 condition: research challenges, strategies, and importance of Core Outcome Set development. BMC Med.

[12] Zheng YB, Zeng N, Yuan K, Tian SS, Yang YB, Gao N (2023). Prevalence and risk factor for long COVID in children and adolescents: a metaanalysis and systematic review. J Infect Public Health.

[13] Aldè M, Di Berardino F, Ambrosetti U, Barozzi S, Piatti G, Zanetti D (2023). Audiological and vestibular symptoms following SARS-CoV-2 infection and COVID-19 vaccination in children aged 5–11 years. Am J Otolaryngol.

[14] Elmas B, Çavdaroğlu PD, Orhan MF, Ay G, Caner I, Tarım A (2021). Evaluation of taste and smell disorders in pediatric COVID-19 cases. Rev Assoc Med Bras (1992).

[15] Panda PK, Sharawat IK, Panda P, Natarajan V, Bhakat R, Dawman L (2021). Neurological complications of SARS-CoV-2 infection in children: a systematic review and meta-analysis. J Trop Pediatr.

[16] Page MJ, Moher D, Bossuyt PM, Boutron I, Hoffmann TC, Mulrow CD (2021). PRISMA 2020 explanation and elaboration: updated guidance and exemplars for reporting systematic reviews. BMJ.

[17] World Health Organization [homepage on the Internet] WHO supports response to new Ebola outbreak.

[18] Lizarondo L, Stern C, Carrier J, Godfrey C, Rieger KL, Salmond S, Aromataris E, Lockwood C, Porritt K, Pilla B, Jordan Z (2017). JBI Manual for Evidence Synthesis.

[19] Abbati G, Attaianese F, Rosati A, Indolfi G, Trapani S (2022). Neurological involvement in children with COVID-19 and MIS-C: a retrospective study conducted for more than two years in a Pediatric Hospital. Children (Basel).

[20] Alzahrani MM, Alaraifi AK, Aldosari LH, Hijazi LO, Alsaab FA (2023). Clinical manifestations of COVID-19 versus other upper respiratory tract infections in pediatric patients. Saudi Med J.

[21] Borch L, Holm M, Knudsen M, Ellermann-Eriksen S, Hagstroem S (2022). Long COVID symptoms and duration in SARS-CoV-2 positive children – a nationwide cohort study. Eur J Pediatr.

[22] Dilber B, Aydın ZG, Yeşilbaş O, Sağ E, Aksoy NK, Gündoğmuş F (2021). Neurological manifestations of pediatric acute COVID infections: a single center experience. J Trop Pediatr.

[23] Elvan-Tuz A, Karadag-Oncel E, Kiran S, Kanik-Yuksek S, Gulhan B, Hacimustafaoglu M (2022). Prevalence of anosmia in 10.157 pediatric COVID-19 cases: multicenter study from Turkey. Pediatr Infect Dis J.

[24] Fang C, Zhou Y, Fan W, Zhang C, Yang Y (2023). Clinical features of febrile seizures in children with COVID-19: an observational study from a tertiary care hospital in China. Front Pediatr.

[25] Flores-Alanis A, Saldaña-Ahuactzi Z, Parra-Ortega I, López-Ramírez P, Salazar-García M, Alemán-García YP (2022). Clinical characteristics of coronavirus disease (COVID-19) in Mexican children and adolescents. Viruses.

[26] Gürkas E, Dünya B, Köken Ö, Demirdağ T, Yilmaz D, Özyürek H (2021). Neurologic manifestations of COVID 19 in children: prospective study in a single center. Ann Indian Acad Neurol.

[27] Hegazi MA, Saeedi FA, Atwah AF, Sayed MH, Albeladi AA, Alyoubi S (2022). Neurological manifestations in pediatric COVID-19 patients hospitalized at King Abdulaziz University Hospital, Jeddah, Saudi Arabia: a retrospective study. Children (Basel).

[28] Kumar L, Kahlon N, Jain A, Kaur J, Singh M, Pandey AK (2021). Loss of smell and taste in COVID-19 infection in adolescents. Int J Pediatr Otorhinolaryngol.

[29] LaRovere KL, Poussaint TY, Young CC, Newhams MM, Kucukak S, Irby K (2023). Changes in distribution of severe neurologic involvement in US Pediatric Inpatients With COVID-19 or multisystem inflammatory syndrome in children in 2021 vs 2020. JAMA Neurol.

[30] LaRovere KL, Riggs BJ, Poussaint TY, Young CC, Newhams MM, Maamari M (2021). Neurologic involvement in children and adolescents hospitalized in the United States for COVID-19 or multisystem inflammatory syndrome. JAMA Neurol.

[31] López-Pérez BJ, Cruz-Chávez DA, Solórzano-Gómez E, Venta-Sobero JA, Tapia-García IA, Toledo-Lozano CG (2022). Neurological manifestations in pediatric patients hospitalized for COVID-19: experiences of the national medical center "20 de Noviembre" in Mexico City. Children (Basel).

[32] Memar EH, Heidari M, Ghabeli H, Pourbakhtyaran E, Haghighi R, Hosseiny SM (2023). Neurologic manifestations of coronavirus disease 2019 in children: an Iranian Hospital-Based Study. Arch Iran Med.

[33] Okur DS (2022). Neurological symptoms and signs associated with COVID-19 in pediatric patients: a single-center experience. Medicine (Baltimore).

[34] Pascarella A, Maglione M, Lenta S, Sciorio E, Mancusi R, Tucci C (2022). Seizures in children with SARS-CoV-2 infection: epidemiological, clinical and neurophysiological characterization. Children (Basel).

[35] Rastogi S, Gala F, Kulkarni S, Gavali V (2022). Neurological and neuroradiological patterns with COVID-19 infection in children: a single institutional study. Indian J Radiol Imaging.

[36] Ray ST, Abdel-Mannan O, Sa M, Fuller C, Wood GK, Pysden K (2021). Neurological manifestations of SARS-CoV-2 infection in hospitalised children and adolescents in the UK: a prospective national cohort study. Lancet Child Adolesc Health.

[37] Riva A, Piccolo G, Balletti F, Binelli M, Brolatti N, Verrotti A (2022). Acute neurological presentation in children with SARS-CoV-2 infection. Front Pediatr.

[38] Rusetsky Y, Meytel I, Mokoyan Z, Fisenko A, Babayan A, Malyavina U (2021). Smell status in children infected with SARS-CoV-2. Laryngoscope.

[39] Salleh HP, Soon IS, Chong VH (2023). Frequency and risk factors for febrile seizures during COVID-19 pandemic waves: an observational study. Eur J Pediatr.

[40] Stephenson T, Pereira SM, Nugawela MD, McOwat K, Simmons R, Chalder T (2023). Long COVID-six months of prospective follow-up of changes in symptom profiles of non-hospitalised children and young people after SARS-CoV-2 testing: a national matched cohort study (The CLoCk) study. PLoS One.

[41] Westbrook AL, Benedit LC, Frediani JK, Griffiths MA, Khan NY, Levy JM (2022). Predictive value of isolated symptoms for diagnosis of severe acute respiratory syndrome coronavirus 2 infection in children tested during peak circulation of the delta variant. Clin Infect Dis.

[42] Yılmaz D, Üstündağ G, Büyükçam A, Salı E, Çelik Ü, Avcu G (2023). A snapshot of pediatric inpatients and outpatients with COVID-19: a point prevalence study from Turkey. Eur J Pediatr.

[43] Romero-Sánchez CM, Díaz-Maroto I, Fernández-Díaz E, SánchezLarsen A, Layos-Romero A, García-García J (2020). Neurologic manifestations in hospitalized patients with COVID-19: the ALBACOVID registry. Neurology.

[44] Favas TT, Dev P, Chaurasia RN, Chakravarty K, Mishra R, Joshi D (2020). Neurological manifestations of COVID-19: a systematic review and meta-analysis of proportions. Neurol Sci.

[45] Cho SM, White N, Premraj L, Battaglini D, Fanning J, Suen J (2023). Neurological manifestations of COVID-19 in adults and children. Brain.

[46] Jin H, Hong C, Chen S, Zhou Y, Wang Y, Mao L (2020). Consensus for prevention and management of coronavirus disease 2019 (COVID-19) for neurologists. Stroke Vasc Neurol.

[47] Ibekwe TS, Fasunla AJ, Orimadegun AE (2020). Systematic review and meta-analysis of smell and taste disorders in COVID19. OTO Open.

